# Ecological assessment of the marine ecosystems of Barbuda, West Indies: Using rapid scientific assessment to inform ocean zoning and fisheries management

**DOI:** 10.1371/journal.pone.0189355

**Published:** 2018-01-08

**Authors:** Benjamin Ruttenberg, Jennifer E. Caselle, Andrew J. Estep, Ayana Elizabeth Johnson, Kristen L. Marhaver, Lee J. Richter, Stuart A. Sandin, Mark J. A. Vermeij, Jennifer E. Smith, David Grenda, Abigail Cannon

**Affiliations:** 1 Department of Biological Sciences, California Polytechnic State University, San Luis Obispo, CA, United States of America; 2 Marine Science Institute, University of California Santa Barbara, Santa Barbara, CA, United States of America; 3 Waitt Institute, La Jolla, CA, United States of America; 4 CARMABI, Piscaderabaai z/n, Willemstad, Curacao; 5 South Florida Caribbean Network, National Park Service, St. John, USVI; 6 Center for Marine Biodiversity and Conservation, Scripps Institution of Oceanography, University of California San Diego, La Jolla, CA, United States of America; 7 Institute for Biodiversity and Ecosystem Dynamics, University of Amsterdam, Amsterdam, The Netherlands; 8 Independent Researcher, Lakeland, FL, United States of America; Leibniz Centre for Tropical Marine Research, GERMANY

## Abstract

To inform a community-based ocean zoning initiative, we conducted an intensive ecological assessment of the marine ecosystems of Barbuda, West Indies. We conducted 116 fish and 108 benthic surveys around the island, and measured the abundance and size structure of lobsters and conch at 52 and 35 sites, respectively. We found that both coral cover and fish biomass were similar to or lower than levels observed across the greater Caribbean; live coral cover and abundance of fishery target species, such as large snappers and groupers, was generally low. However, Barbuda lacks many of the high-relief forereef areas where similar work has been conducted in other Caribbean locations. The distribution of lobsters was patchy, making it difficult to quantify density at the island scale. However, the maximum size of lobsters was generally larger than in other locations in the Caribbean and similar to the maximum size reported 40 years ago. While the lobster population has clearly been heavily exploited, our data suggest that it is not as overexploited as in much of the rest of the Caribbean. Surveys of Barbuda’s Codrington Lagoon revealed many juvenile lobsters, but none of legal size (95 mm carapace length), suggesting that the lagoon functions primarily as nursery habitat. Conch abundance and size on Barbuda were similar to that of other Caribbean islands. Our data suggest that many of the regional threats observed on other Caribbean islands are present on Barbuda, but some resources—particularly lobster and conch—may be less overexploited than on other Caribbean islands. Local management has the potential to provide sustainability for at least some of the island’s marine resources. We show that a rapid, thorough ecological assessment can reveal clear conservation opportunities and facilitate rapid conservation action by providing the foundation for a community-driven policymaking process at the island scale.

## Introduction

Throughout the Caribbean, the condition of coral reef ecosystems has been in decline for several decades [1,2], and coral reef scientists have produced a vast literature documenting ecosystem decline and evaluating the putative drivers [2–6]. While the relative importance of specific drivers may vary by location, they include global factors such as climate change and ocean acidification, regional factors such as disease, and local factors such as eutrophication, coastal development, coastal runoff, and sedimentation (together referred to as ‘land-based sources of pollution,’ or LBSPs), as well as overfishing [1,3,7–9]. Understanding the relative importance of global, regional, and local factors is critical for management, because resource managers are only able to control or mitigate local factors, and usually only a subset of these. Local management strategies include changing watershed management to reduce LBSPs, changing fishery management in an attempt to improve sustainability of fisheries, or implementing spatial management such as marine protected areas (MPAs) in an attempt to control multiple factors at once [10–14]. Despite recent efforts to improve local management, the condition of many Caribbean coral reef ecosystems has continued to decline ([10,15,16], but see [17,18]).

Identifying specific drivers of decline has been a major priority for conservation and applied research in the region. Using a synthesis of long-term data from around the Caribbean, Jackson et al. [1] found that patterns of change in coral cover varied in different locations, suggesting that the causes of ecosystem change may also vary spatially. They suggest that local factors, particularly fishing, may have been directly or indirectly responsible for some of the observed declines. While regional and global factors clearly contribute to declines in the conditions of coral reef systems worldwide, managing local factors effectively may be able to reverse or slow declines in some situations [9,10,15,19]. Similar work examining predatory and herbivorous reef fish assemblages throughout the Caribbean found that human population size was inversely correlated with fish abundance [4,12]. These results suggest that local factors influence fish abundance, and other recent work suggests that a suite of social factors such as governance and the strength of local institutions can have strong impacts on the condition of local reefs ([20]).

While continued declines in the condition of Caribbean coral reef ecosystems are troubling, it is encouraging that some of the decline may be related to local factors, which are easier for local communities to address than factors that are regional or global in scope. Still, developing appropriate management strategies for local factors requires local information, and the lack of scientific information in many locations has in part hindered implementation of effective management strategies. Further complicating the situation for managers is the fact that different research programs often use different metrics to assess aspects of ecosystem health [1]. The most common measure of coral reef condition is percent cover of live coral, but there are many other metrics of coral condition and ecosystem condition that relate to the functioning and recovery potential of reefs (e.g., percent cover of reef building organisms and fleshy algae, ratio of calcifying to non-calcifying organisms, rates of productivity and recruitment, abundance of key grazers, and abundance, size, or biomass of fishery species [3,4,10,14,16,21,22]). Therefore, it can be challenging for managers who want to use science-based management tools to identify and use the best metrics for a given management scenario. One solution to this challenge is for governments and managers to collaborate with scientists to conduct a rapid, thorough, resource assessment at the island-wide scale using the best available scientific methods.

The island of Barbuda in the Eastern Caribbean is an example of a location where the local community has a strong desire to manage ecosystems sustainably but has lacked the capacity to collect the necessary scientific information to achieve this. As part of the Blue Halo Initiative of the Waitt Institute, a collaborative marine conservation and marine zoning project began on Barbuda in 2013. Initial consultations with Barbudans helped identify data needs and data gaps for an ecological assessment of the shallow water marine ecosystems around the entire island. The goals of this study were to: 1) measure a range of ecological indicators, including the abundance and size structure of the key export fishery species (i.e., spiny lobster and conch), the abundance, biomass, and size structure of the reef fish assemblage, and the percent cover of key benthic functional groups (e.g., corals, macroalgae, and turf algae), 2) compare these data to other locations in the Caribbean, and 3) use these data to make management recommendations. We made it a priority to include local fishers and resource managers in the research, to ensure that local ecological knowledge was integrated with scientific knowledge and reflected in the conclusions. This also helped build stakeholder trust in the final results. The Blue Halo Initiative provided SCUBA certifications and equipment to facilitate this collaboration, which also enabled Barbudans to conduct follow-up surveys using best-available scientific methods.

After receiving the findings from this study, the local governing council used this information in a community-driven process to design and implement a network of marine protected areas around the island of Barbuda. The data presented here describe the state of Barbuda’s marine ecosystems in 2013, including opportunities for sustainable management of key export fisheries. In September 2017, during the preparation of this manuscript, Category 5 Hurricane Irma made landfall on Barbuda, destroying the majority of buildings on the island and displacing all residents. It also likely had significant negative impacts on many of the island’s marine ecosystems. Still, these results provide a baseline for future work evaluating recovery from storms and the potential effectiveness of Barbuda’s management actions. Finally, our approach demonstrates the broader utility of a rapid, thorough, island-wide marine resource and ecological assessment as the foundation for a comprehensive, community-driven policymaking process.

## Methods

### Setting and history of Barbuda

**Fig 1:**
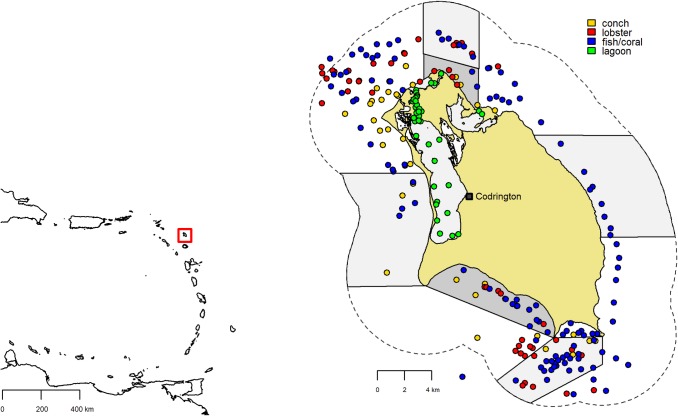
Map of Barbuda. Map of survey sites and types of surveys conducted, with newly-implemented no-take zones marked in light gray and zones where net fishing is prohibited marked in darker gray. The 3 nm territorial waters are indicated with a dashed line. Codrington Village is marked along the eastern edge of Codrington Lagoon. The inset map shows the Eastern Caribbean, with Barbuda marked with a red box.

Barbuda is a low-lying coral limestone island in the northeastern corner of the Windward Islands of the Lesser Antilles in the Caribbean. It is the northernmost island of the country of Antigua and Barbuda, located at approximately 17.64 N, 61.82 W (Fig 1). Barbuda is exposed to the north and east to the open Atlantic and therefore to major ocean swells from these directions. Depth increases quickly to the north and east, reaching depths of >100 m within a few km, and depths of >1000 m within 20 to 40 km. However, the south and west sides of the island have wide, shallow platforms of only 20–25 m depth, which extend at least 10 km from the island. The maximum depth between Barbuda and Antigua, located 50 km to the south, is only 30 m. The island covers 160 km^2^, and the vast majority is <10 m in elevation. Barbuda also contains Codrington Lagoon, managed as a national park, which covers 18 km^2^. The lagoon is 8 m at its deepest point but the majority of the area is <3 m depth. The benthos in the lagoon is a mix of sand, macroalgae, and seagrass, all surrounded by mangroves, and water enters and exits the main lagoon through a narrow, 3 km-long channel and through a few outer lagoons.

Before the hurricanes of September, 2017 (Irma and Jose), the human population of Barbuda was approximately 1,800, with most living in Codrington Village, situated on the east shore of Codrington Lagoon (Fig 1). While Barbuda once had sugar cane plantations, there is little active agriculture today, and the island has limited tourism or tourism infrastructure. Barbuda is seasonally dry, and with the small human population concentrated around Codrington, there is minimal terrestrial runoff, especially to the east, south, and west.

At the large scale, Barbuda’s nearshore ecosystems are typical of many Caribbean islands; inshore areas are dominated by seagrass in shallow (<5 m) waters. Seagrass gives way to low-relief hardbottom mixed with patch reefs further from shore. The north and east shores are fringed by forereefs, and the south shore has a series of deeper large (>1000 m^2^) patch reefs. The west shore is dominated by low-relief hardbottom with patch reefs that extend at least 10–15 km from the island. However, Barbuda generally lacks the high-relief spur and groove habitats common on many other Caribbean islands.

Caribbean spiny lobster (*Panulirus argus*) is the most economically important fishery species on Barbuda [23]. Lobsters are targeted by divers and trap fishers working from small boats as far as 20 km or more from the island. Legal size is 95 mm carapace length, and lobsters are primarily exported to the French islands in the Lesser Antilles, bringing in an estimated $2.5 million per year to the local economy [23]. Undersized lobsters are caught at an unknown rate and generally consumed locally. Queen conch (*Lobatus gigas*) is the second most important fishery species, also targeted by divers or snorkelers from small boats, usually in the shallow, inshore seagrass areas. Legal conch must be at least 180 mm in length from the apex to the siphonal notch and must have a flared shell lip, which usually develops around sexual maturity [24]. However, active shell middens around the island often contain some undersized conch lacking the flared lip, indicating recent take of sublegal conch. Reef fish are caught for local consumption, using hook and line, spears, and gill nets. There are limited data on landings for fish.

We restricted most surveys to within 3 nautical miles (5.56 km) of Barbuda; waters in this area are Barbudan territorial waters and are under the jurisdiction of the governing council of Barbuda. All surveys were conducted between 15–25 April 2013. Because our work was exclusively based on field surveys, we made no collections of any species, including endangered or protected species. No collecting permits were required from the Barbudan government. All surveys were conducted from private vessels (see acknowledgments) or from vessels owned by the Codrington Lagoon National Park, with permission and collaboration from the staff of the National Park.

### Survey methods

#### Lobster

Caribbean spiny lobsters are often patchy in their distribution; this is particularly true around Barbuda, where lobsters are often found under specific ledges and in fissures in low-relief hardbottom rather than uniformly distributed on reefs. To address this surveying challenge, we focused our efforts on collecting data on relative abundance, size structure, and sex ratios. For lobster surveys, we paired professional scientists with local Barbudan lobster divers, because local fishers are much more skilled at finding and catching lobsters. For the initial surveys, we randomly selected survey locations. However, because of the patchy distribution of lobsters, most of these surveys recorded zero lobsters. Therefore, we modified the protocol to allow fishers to select any fishing sites within Barbudan territorial waters, which made estimation of density impossible but produced data to examine size structure and sex ratio across areas of known lobster habitat.

We determined the sex and carapace length (CL; in mm) of each lobster captured, and classified each lobster as at or above the legal harvest size (>95 mm CL in Antigua and Barbuda; hereafter ‘legal lobster’) or below the legal size (hereafter ‘sublegal lobster’). At sites where we observed juvenile/sublegal lobsters but were unable to capture and measure them, we estimated numbers and sizes. In total, we surveyed 52 sites for lobster abundance, sex ratio, and size structure in depths ranging from 1 to 18 m. Because the patchy nature of lobster distribution led us to prioritize collecting data on relative abundance, sex ratio, and size structure in places where lobsters are known to occur, we could not convert our abundance data to island-wide density (i.e., numbers per unit area). Instead, we used these data to visualize the occurrence of lobsters around Barbuda. We used a t-test to examine sex-based differences in size structure.

#### Conch

Sites for conch surveys were randomly selected from sand and seagrass habitats. We navigated to the pre-selected site using GPS and we deployed a weighted line to mark the site. The weight served as a central point from which divers surveyed four belt transects of 50 m x 4 m, beginning 1 m from the central point to eliminate overlap between transects. Transects were oriented in cardinal directions (N, S, E, W), and the four transects covered a total area of 800 m^2^ per site. All conch within 2 m of either side of each transect were counted and measured for total length (in cm, from tip of the spire to the end of the siphonal canal). Divers classified conch as adults or juveniles based on the presence of a flared lip at the opening of the shell [25]. We conducted conch surveys at 35 sites in depths from 1 m to 15 m.

#### Fish

Sites for fish surveys were selected haphazardly using a combination of satellite imagery and local knowledge of the location of hard-bottom habitats. Our goal was to distribute sampling effort as across habitats and regions of the island, focusing on hard-bottom habitats. Coral and benthic surveys (see following section) were conducted concurrently, with one fish diver and one benthic diver per site. Fish surveys were conducted using the stationary point count method [26,27]. Briefly, divers descended to the bottom and the fish survey diver haphazardly selected a point on the bottom which became the center for a cylinder of 7.5 m radius. The diver listed all of the species present inside the cylinder and estimated total numbers and sizes one species at a time. After estimating abundance for a given species, the fish survey diver ignored that species for the remainder of the count. New species seen for the first time after 5 minutes were noted as such. Mobile species seen after 5 minutes were excluded from density calculations, while sedentary or cryptic species seen between 5 and 10 minutes were included in density calculations. At the end of the count, the fish survey diver recorded habitat type (i.e., continuous or patch reef), maximum vertical reef in the cylinder, and depth. Sites were assigned to a habitat class based on the combination of habitat type, relief (low relief: <1.5 m, medium relief: 1.5–3 m, high relief: >3 m), and depth (shallow: <6 m, mid: 6–18 m, deep: >18 m)[26]. After completing one count, the fish diver swam approximately 15 m from the center of the first cylinder, and completed a second count using the same procedure. The two counts were averaged to obtain an estimate of the density of all species at the site, in units of number of individuals per cylinder (177 m^2^). We conducted these surveys at 116 sites at depths that ranged from 2 m to 25 m. In total, we recorded data for 110 diurnally-active, reef-associated species.

For each fish species, we calculated biomass using published length-weight relationships [28,29], and computed mean density and biomass per site for each species (S1 Table). We computed per-site estimates of biomass for several sets of species (e.g., total fish biomass, biomass of snappers and groupers, and parrotfish biomass) at each site, as well as overall frequency of occurrence for each species. We used non-metric multidimensional scaling of density data to visualize variation in community structure in different regions and different habitats around the island using the function ‘metaMDS’ in the ‘vegan’ package in R 3.3.0 [30]. For this analysis, we restricted the species list to those species present at >10% of sites.

#### Coral and benthic cover

Surveys for coral and benthic cover were conducted concurrently with fish surveys and focused on percent benthic cover. The benthic survey diver descended with the fish survey diver and laid out a 25 m transect from a haphazardly selected location at least 7.5 m from the fish diver to avoid interfering with the fish survey. The benthic diver then took a photograph of the benthos every 2 m using a camera mounted on an 80 cm long PVC pole, ensuring that the camera captured the same total surface area in each photograph. After completing the first transect, the benthic diver laid a second 25 m transect in the opposite direction and repeated the process of taking photos as described above. This process yielded 24 photographs per site. In total, the ecological assessment team surveyed the benthic communities at 108 sites around Barbuda.

We used the computer software Photogrid (C. Bird, University of Hawaii, Botany Dept.) to estimate percent cover of benthic organisms. The primary space-occupying organism was identified under each of 25 randomly-placed points for each photograph. Organisms were identified to the lowest taxonomic level possible or to functional group as follows: all stony corals were identified to species, many algae, soft corals, and sponges were identified to genus, and other organisms were placed in functional group categories (e.g., turf algae and crustose coralline algae [CCA]) [31]. The epilithic matrix of turf algae and fine sediment was dominant in nearly all sites around Barbuda and was classified as ‘turf.’ We excluded points over holes, shadows, or unidentified organisms from the analyses. In total, we analyzed 71,979 points in 2,854 benthic photographs. We also examined the relationship between different benthic groups and fish assemblages using linear regression (using the function ‘cor.test’ in the ‘stats’ package in R 3.3.0 [30]).

#### Lagoon

We selected sites within the lagoon and the channel haphazardly, with an emphasis on the mangrove channel. At each site, we deployed four 15 m transects. In areas with mangroves (e.g., the channel at the lagoon entrance), transects followed the mangrove edge. In areas in the middle of the lagoon, transects were deployed in a straight line from a haphazardly selected starting point. Transects were surveyed by a pair of snorkelers. Along each transect, one snorkeler identified, counted, and estimated sizes for each fish and lobster in a belt 2 m wide (1 m on either side of the tape). The second snorkeler took downward-facing photos of the bottom every meter, using a similar approach to that of the benthic surveys. The data for all four transects were averaged to generate a single estimate of the benthic community at each site. We surveyed 31 sites in the lagoon and channel.

### Results and discussion

#### Habitat

Much of the hardbottom reef around Barbuda are low-relief carbonate flats, often covered in a matrix of sediment and algal turfs (S1 Fig). These low-relief habitats contain occasional cracks or fissures of one to several meters in diameter and 0.5–1 m deep. These fissures were relative ‘hot-spots’ for fish and lobster, identified both through our own observations and through consultation with local fishers. These fissures were also uncommon and patchily distributed, making it effectively impossible to quantify their abundance and distribution. The broader low relief habitats of Barbuda are present in other areas of the Caribbean, but they are the dominant habitat type around Barbuda. Of the 71 forereef sites surveyed, 55 (77%) were low-relief hardbottom habitats. The remaining sites surveyed were more typical Caribbean patch reef habitats (S1 Fig). Despite apparent recent (year-to-decade scale) mortality of live coral tissue (see section Coral, below), these habitats retained vertical relief from standing coral skeletons, and 35 of 45 (78%) of patch reef habitats surveyed were medium or high relief.

#### Lobster

**Fig 2:**
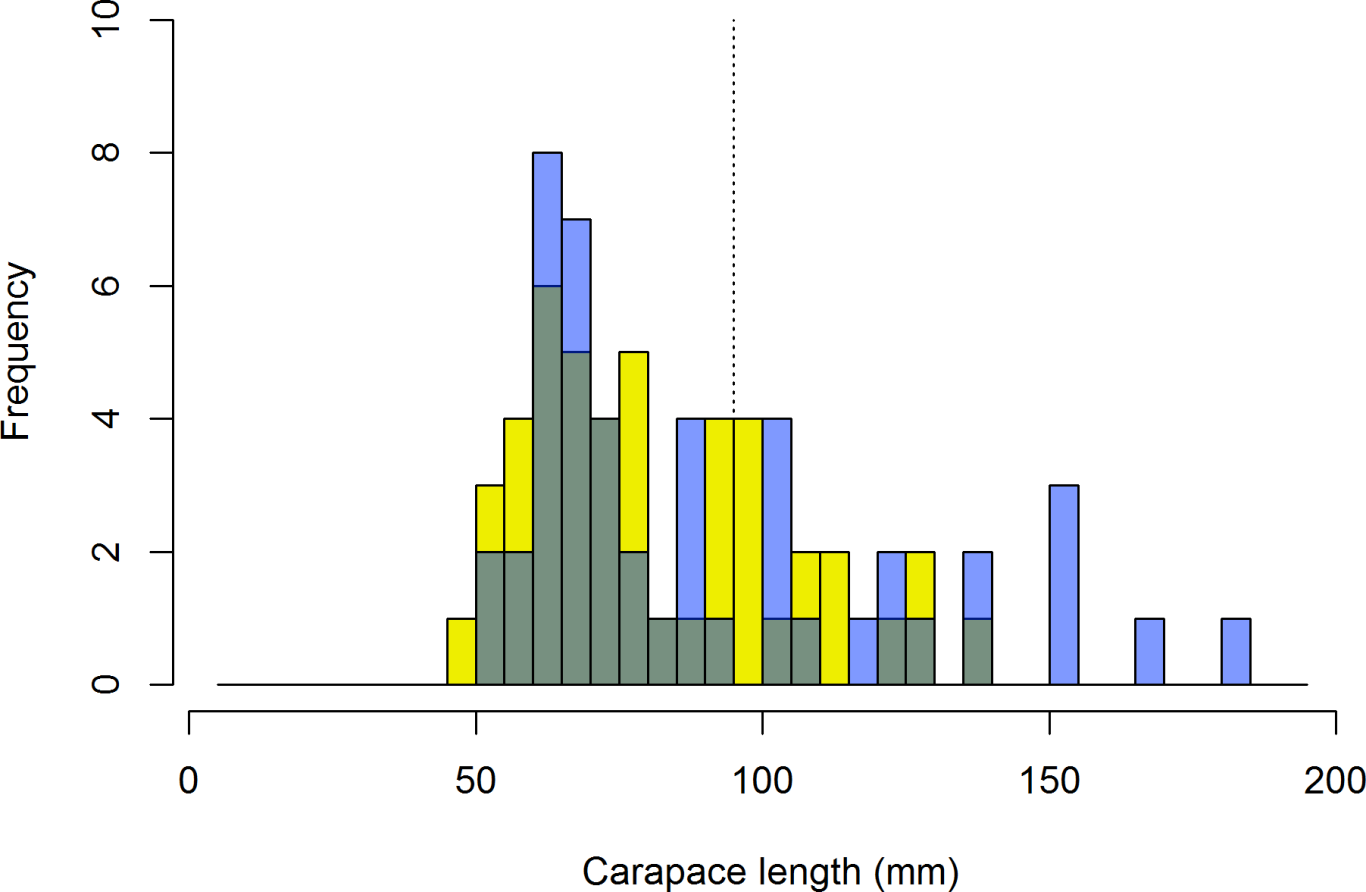
Size frequency histogram of lobster data. Light blue bars represent males, yellow bars represent females, and gray bars represent overlap. The vertical dashed line is at 95 mm carapace length, the minimum legal size in Barbuda.

Consistent with the patchy nature of lobster distribution, lobsters were only present at 13% of the randomly selected sites surveyed. Considering only the sites selected by Barbudan fishers, lobsters were present at 65% of sites, with 0.8 ± 0.3 legal and 2.1 ± 0.8 sublegal lobsters per site (mean ± SE reported throughout the manuscript; these results exclude an outlier site with an aggregation of >70 sublegal lobsters; S2 Fig). Of the lobsters we caught and measured, 32% were of legal size. The sex ratio (females:males) of measured lobsters was exactly 1 (n = 94), similar to that of other studies[32,33]. Although size was not significantly different between sexes for all individuals (91 ± 4.9 mm for males, 82 ± 3.3 mm for females; t-test: t = 1.6, df = 92, p>0.10), carapace length of the legal males was larger than that of the legal females (mean size ± SE: 131 ± 6.2 mm for legal males and 112 ± 3.6 mm for legal females; t-test: t = 2.5, df = 27, p<0.02; Fig 2).

Inside Codrington Lagoon (see Lagoon section for description of methods), we observed 1.39 ± 0.7 lobsters per site (excluding a single nonrandomly selected site with a known high abundance of lobsters; we counted 52 lobsters at this site), with an average carapace length of 51 ± 0.8 mm (n = 108), and a maximum of approximately 80 mm. No legal lobsters were observed in the lagoon, consistent with lagoon surveys conducted in 1973, suggesting the lagoon has either been primarily functioning as a nursery habitat and/or is experiencing high fishing pressure [34].

Maps of the distribution of legal and sublegal lobsters illustrate their distribution (S2 Fig). Legal lobsters were most common between 5–20 m depth on the north and south shores, but were virtually absent from inshore patch reefs. Sublegal lobsters were present in moderate numbers (1–12 individuals per site) between 5–20 m depth, but two of the three sites with the highest density of sublegal lobsters were close to shore, and all three were near the opening to the lagoon, consistent with the idea that the lagoon provides important nursery habitat for lobsters (S2 Fig). Our surveys corroborated reports from local fishers that lobsters are patchily distributed in Barbuda. This may be partly a result of the patchy nature of the habitat around the island, the fact that lobsters are often gregarious, or the heavier levels of fishing pressure in some locations.

We observed some lobsters around Barbuda that were much larger than those observed in other areas of the Caribbean and subtropical Atlantic [35]. For example, lobsters in areas open to fishing in the Florida Keys, where fishing pressure is very intense, reached maximum sizes of approximately 90 and 100 mm CL for females and males, respectively [36]. Maximum sizes were larger in marine reserves in the Florida Keys (110 and 135 mm CL for females and males, respectively), but still smaller than the largest we observed around Barbuda (140 and 183 mm CL for females and males, respectively).

The maximum size of lobsters around Barbuda has remained more or less unchanged in the past 40 years; Peacock (1974) found a maximum length of 170 mm in the early 1970s. However, only 32% of the lobsters that we captured and measured were legal, likely a much smaller fraction than the “great majority” of lobsters >90 mm CL that were observed in Barbuda 40 years ago [34]. This decline in abundance of legal lobsters relative to sublegal lobsters suggests that fishing is removing many large individuals. In addition, the fishery 40 years ago was conducted near to shore by free diving, and today most of the Barbuda lobster fishery uses scuba at sites that may be >5–10 km from shore. Still, the presence of large individuals and the fact that the maximum size is similar to what it was 40 years ago suggest that the lobster population around Barbuda has not been as overexploited as many other locations in the Caribbean, and likely has the potential to increase with changes in management.

#### Conch

Conch were present at 86% of sites, with adult conch present at 53% of conch survey sites, and juveniles present at 71% of sites. Densities averaged 0.29 ± 0.12 adult conch per 100 m^2^ and 0.96 ± 0.30 juvenile conch per 100 m^2^ (mean ± SE). We excluded one outlier site (>25 juvenile conch per 100 m^2^) from these mean density calculations, since it may have been an aggregation [37,38], but we included it on the map (S3 Fig). Juvenile conch density increased to 1.73 ± 0.82 per 100 m^2^ (mean ± SE) when including this site.

For comparison, current densities of adult conch around Barbuda are similar to those in locations considered “low density” such as Barbados (less than 0.1 adults per 100 m^2^, [39]) and lower than in locations such as Belize (2.4 adults per 100 m^2^; [40]) and the Bahamas (0.6 to 1.3 adults per 100m^2^; [41]). As with the lobster data, there was no clear pattern in the spatial distribution of juvenile or adult conch around the island. Two inshore sites along the south shore had higher juvenile abundance, and these same two sites were among those with the highest adult abundance, observations that are consistent with conch aggregations in other parts of the Caribbean [39,37].

**Fig 3:**
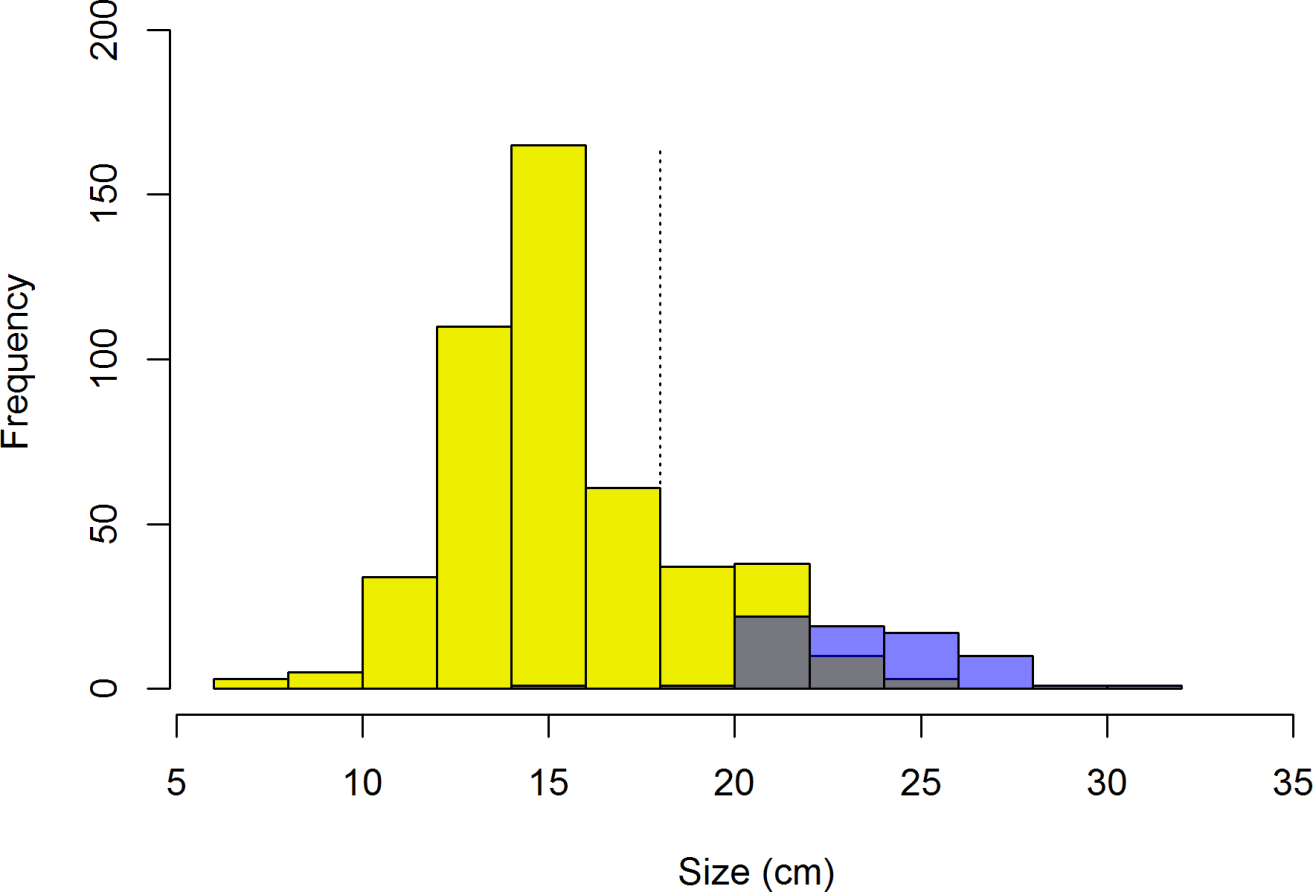
Size distribution of conch in Barbuda. Yellow bars represent juvenile, nonreproductive conch (no flared lip), blue bars represent adult conch (with flared lip), gray represents overlap. The dashed line at 18 cm indicates the legal size of capture for conch in Barbuda.

The largest observed conch was 32 cm total length, but only 13.3% of conch were adults (Fig 3); a similar proportion of juveniles was found in heavily-fished areas of Antigua in 1999 [42]. Similar to the findings of Tewfik et al. [42], we conclude that this low proportion of adults is most likely an indication of fishing pressure. In comparison, some Caribbean locations have high densities of conch and high percentages of adults (e.g., St. Lucia, 1.2 adults per 100 m^2^ and 51% adults; [43]). Although a high percentage of juveniles could be an indication of recent recruitment, our observations more closely resemble those from locations where conch are considered overfished (e.g., Barbados, 0.1 adults per 100 m^2^ and 8% adults; [39]).

There are still some large conch around the island, but they are rare. This finding, combined with relatively low densities and low proportion of adults, suggests that fishing pressure has significantly reduced the adult population. Similar reductions in density and size of adults have resulted in Allee effects in conch populations in other locations [44]. Therefore, increasing the population of reproductive conch will be essential to recovering conch stocks around Barbuda.

Based on discussions with local fishers and comparisons with conch populations elsewhere in the Caribbean, the abundance of conch in Barbuda appears to have declined significantly over the last several decades [40,41]. However, without information on historical densities we cannot determine the extent to which this lower conch density is natural versus a result of fishing, but we suspect fishing plays a major role. Conch are still present at most sites and we observed many juvenile conch (up to 25 per 100 m^2^ at the highest density sites), which suggests that recruitment is ongoing with potential for populations to increase under effective management.

#### Fish

In general, fish abundance and biomass were low around most of the island compared to other locations in the Caribbean [1]. Like many Caribbean locations, larger predators were rare [3,4]; for example, Nassau grouper (*Epinephelus striatus*) and mutton snapper (*Lutjanus analis*) were present at 7% and 5% of sites, respectively, and no other large snapper or grouper species were observed. Smaller groupers such as red hind (*Epinenphelus guttatus*) were more common, present at 44% of sites. Similarly, large parrotfish were nearly absent; rainbow parrotfish (*Scarus guacamaia*) were observed at only two sites (1.7%), while midnight (*Sc*. *coelestinus*) and blue parrotfish (*Sc*. *coeruleus*) were not observed. Most other reef-associated parrotfish species (*Sc*. *taeniopterus*, *Sc*. *vetula*, *Sparisoma rubripinne*, and *Sp*. *viride*) were present at 25–35% of sites, with the exception of the more abundant redband parrotfish (*Sp*. *aurofrenatum*; 90% of sites), and the rare redtail parrotfish (*Sp*. *chrysopterum*; 6% of sites).

**Fig 4:**
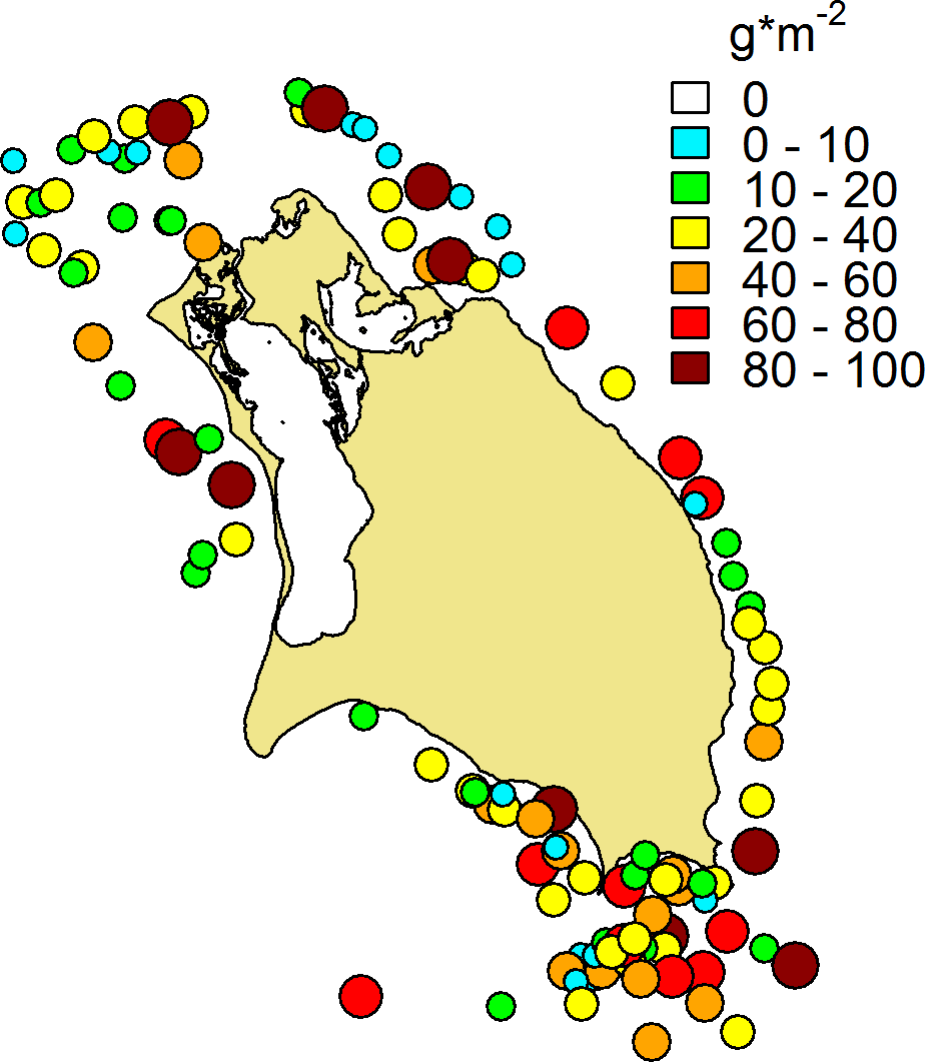
Total fish biomass. Spatial distribution of the total biomass of fish communities around Barbuda. Colors and sizes of symbols indicate bins of biomass density, in units of g m^-2^.

Across all fish survey sites, the total mean biomass was 33 ± 2.3 g m^-2^ (Fig 4). Grouper biomass was 2.3 ± 0.30 g m^-2^, snapper biomass was 1.2 ± 0.28 g m^-2^, and parrotfish biomass was 7.1 ± 0.62 g m^-2^ (S1 Table). These values are lower than much of the rest of the Caribbean, which averages 10–15 g m^-2^ for parrotfish and groupers [1]. Like parrotfish, surgeonfish are important grazers; their biomass averaged 6.4 ± 0.57 g m^-2^, much lower than in some other Caribbean locations, such as Bonaire and Saba, but higher than in some heavily-fished islands, such as Jamaica [45].

Spatial patterns of fish biomass varied among different trophic groups. Total fish biomass showed no clear patterns by location, with both higher and lower biomass sites scattered around Barbuda (Fig 4). In addition, there were no significant differences in total biomass between habitats, depths, or the combination of habitat and depth (ANOVA; p > 0.1 in all cases). However, multivariate plots revealed differences in community structure in different habitats (PERMANOVA; F_10,115_ = 4.4, p<0.001, S4 Fig). Biomass of predatory groupers (Serranidae) and snappers (Lutjanidae) was generally highest on the exposed areas on the east shore of the island (which is less accessible to fishing), with a few higher biomass sites on inshore patch reefs on the south shore (S5 Fig). In contrast, biomass of herbivorous parrotfish (Labridae: Scarinae) and surgeonfish (Acanthuridae) was generally low in the exposed areas along east and north shores, and highest in the patch reefs on the south, west, and northwest shores (S5 Fig).

Based on local ecological knowledge garnered from interviews with local fishers and islanders, as well as published historical information from the Caribbean [1], the abundance of many species of fish appears to have declined around Barbuda. Still, there are some encouraging signs. Nassau groupers, which once dominated reefs and fisheries across the wider Caribbean, have been severely depleted throughout the region, in part because of the ease of fishing their spawning aggregations [46], and they are now virtually absent around many Caribbean islands [47,48]. However, they are still present (though rare) around Barbuda, and we observed a few large individuals. In addition, lionfish (*Pterois volitans*) were present in our fish surveys at only one site, suggesting that the ecological impacts from these invasive predators may be less severe than those observed in other locations [49,50].

**Fig 5:**
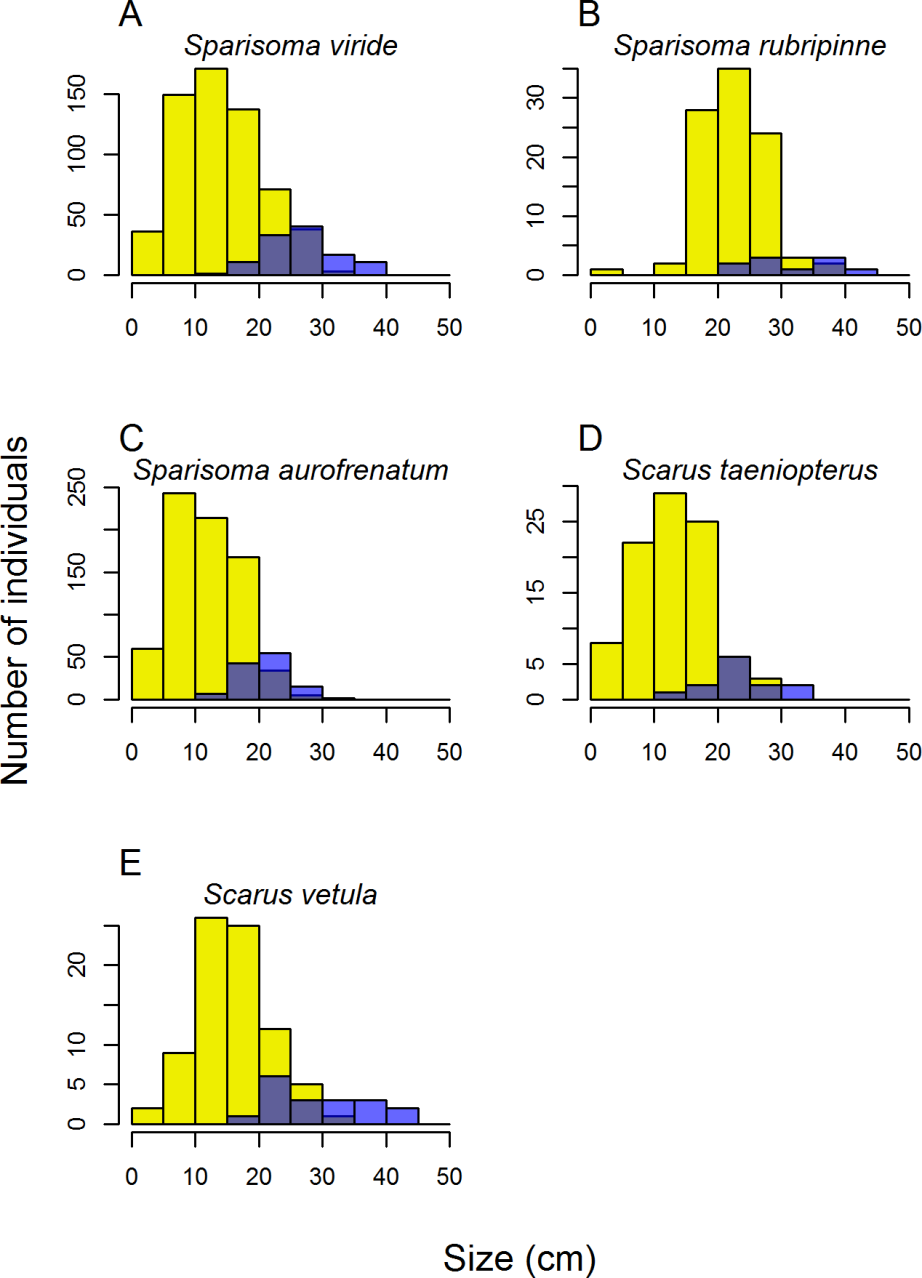
Size-frequency plots for the five most common species of large parrotfish around Barbuda. Gray bars represent juvenile and female fish, blue bars represent terminal phase males, gray represents overlap. Note that y-axis scale changes with each species.

The largest Nassau grouper and mutton snappers observed were 70–75 cm in length. The more common red hind ranged in length from 10–40 cm, averaging 24 ± 0.63 cm, which is smaller than what has been observed in some other Caribbean locations (e.g., US Virgin Islands, 35–40 cm; [51]). Parrotfish size distributions were skewed toward smaller individuals, but initial phase individuals (i.e., juveniles and females) generally ranged from 5–30 cm, with terminal (male) phase individuals ranging from 15–40 cm (Fig 5). Sex-specific size-frequency plots for parrotfish revealed that a relatively small proportion of parrotfish (10–18%) are males (Fig 5). However, because these males are larger, they made up 41% of the total parrotfish biomass. In other locations with lower fishing pressure, such as the Central Pacific, males can make up 30% or more of the individuals [52], but there are few comparable data from elsewhere in the Caribbean [53].

Parrotfish abundance was also relatively low compared with the rest of the Caribbean [1]. Based on our conversations with local community members, parrotfish were much more abundant in Barbuda even as little as five years ago. However, fishers—including those from other islands—have increasingly targeted parrotfish with gill nets and spearguns, particularly in the patch reef habitats where parrotfish are most abundant. It is these habitats where the positive effects of parrotfish grazing are likely to have the greatest benefits to corals by reducing the abundance of algae that compete with both adult and recruiting corals for space [54]. In addition, larger parrotfish may have greater grazing impacts than smaller individuals of the same species [55,56], but the size structure of parrotfish in Barbuda is skewed toward smaller individuals, with few large individuals above 25 or 30 cm (Fig 5).

#### Corals and benthic communities

**Fig 6:**
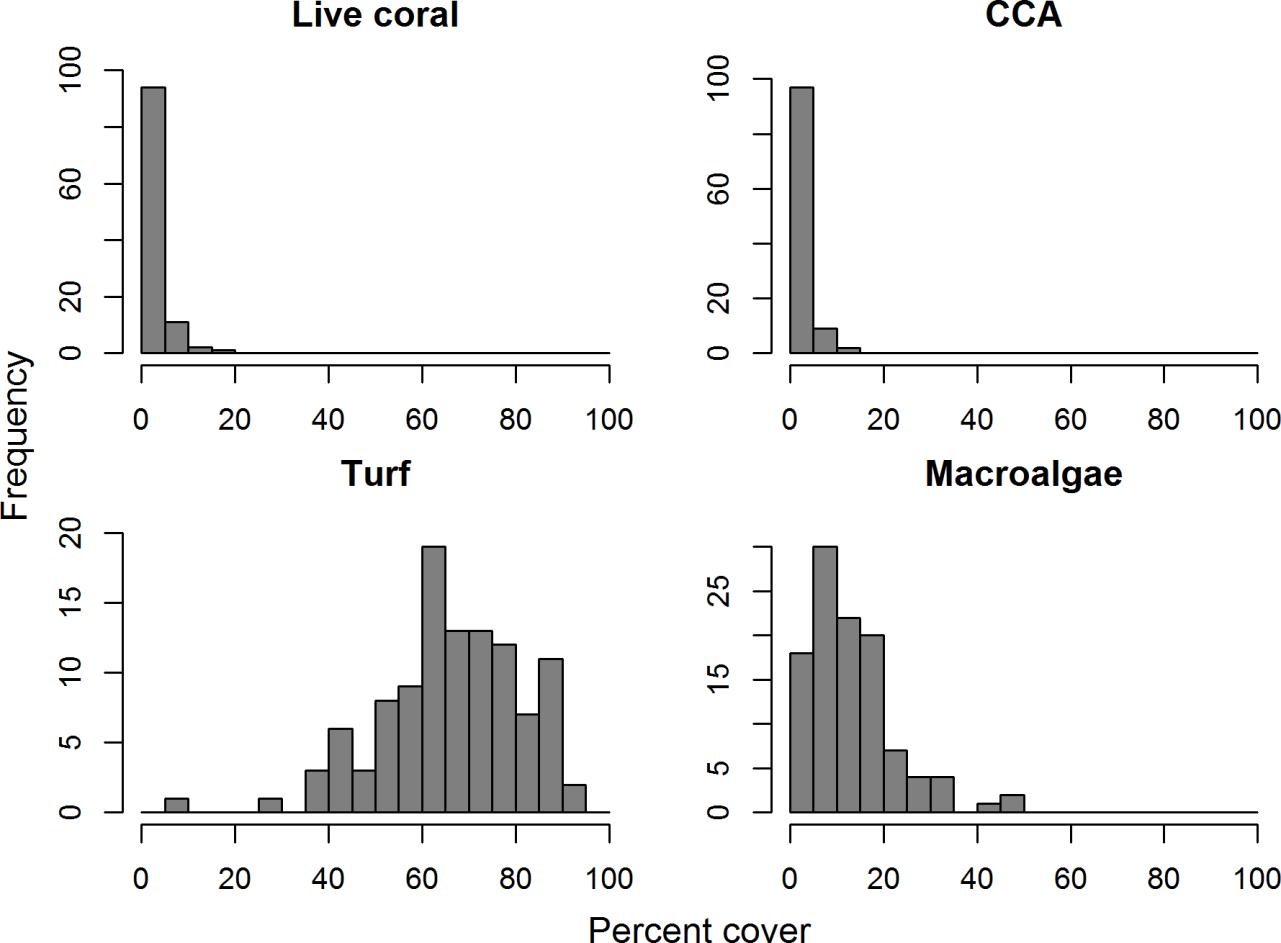
Frequency distribution for percent cover of four major benthic groups. Groups include: Live coral, CCA (crustose coralline algae), Turf algae, and Macroalgae. Note that y-axis scale changes with each group.

We observed 27 species of hard coral around Barbuda. Island-wide, mean live coral was 2.6 ± 0.3% (Fig 6). Of all sites surveyed, the maximum coral cover observed was 16%, but only three sites in total had more than 10% live coral cover. Overall, even the three most common coral species in our surveys, *Porites astreoides*, *Porites porites*, and *Siderastrea siderea* all averaged less than 1% live cover across all sites. The three species in the *Orbicella annularis* complex, an important group of reef-building corals in the Caribbean, had a combined average below 0.6% live cover. The once-dominant species *Acropora cervicornis* and *Acropora palmata* were extremely rare; live *A*. *cervicornis* was present in only two photoquadrats (out of more than 2850 total photoquadrats across all sites), and live *A*. *palmata* was not observed in any photoquadrats.

**Fig 7:**
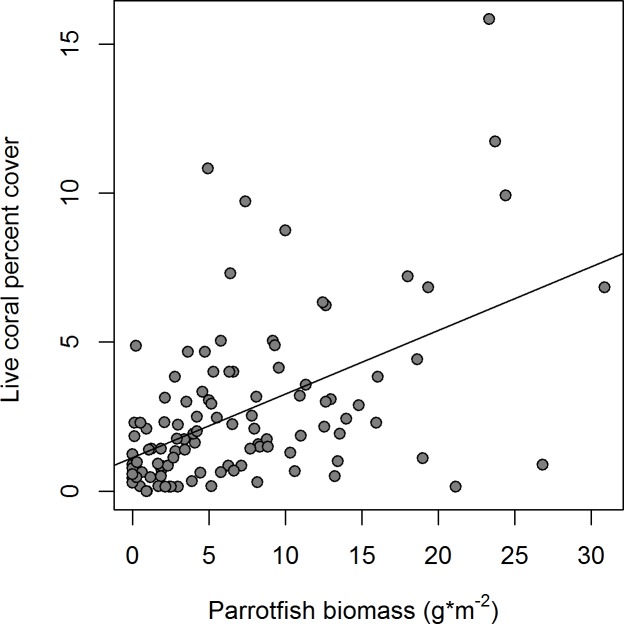
Scatterplot of percent live coral and parrotfish biomass. Data are from 108 sites around Barbuda (r^2^ = 0.25, p < 0.001).

The benthic cover of crustose coralline algae (CCA), another important component of reef calcification [57], was low, averaging only 2.0 ± 0.3%, with a maximum cover of 13%. In contrast, turf and macroalgae were much more abundant on Barbuda’s reefs, with mean cover of 66 ± 1.5% and 13 ± 0.9%, and a maximum cover of 92% and 50%, respectively (Fig 6). We examined the relationship between many of these benthic groups and the relationships between benthic groups and the abundance of several groups of fish. While most of these relationships were non-significant (S6 Fig), there was a highly significant, positive relationship between parrotfish biomass and cover of live coral (r^2^ = 0.25, p < 0.001; Fig 7). However, we cannot determine whether greater abundance of parrotfish is a factor that maintains higher coral cover in certain locations, greater coral cover creates habitat for parrotfishes, or whether both groups are responding to other habitat variables.

Spatially, the sites with the highest coral cover were clustered in the patch reefs along the south shore and (to a lesser extent) along the west shore of Barbuda (S7 Fig). The inshore patch reefs along the north shore had low coral cover in general. The lowest cover overall occurred primarily in the deeper areas outside the forereef, especially in exposed areas of the north and east shores. At all shallow patch reef and back reef habitats, there was clear evidence of recently living coral; many of the skeletons (especially those of *A*. *palmata*) were still standing, intact, and largely covered in fleshy algae.

Island-wide coral cover (2.6 ± 0.27%; mean ± SE) was much lower than the Caribbean average of ~17% [1]. However, many of the forereef survey sites were low relief habitats with little three-dimensional structure. It is possible that these areas have not had high coral cover for decades, centuries, or possibly longer. However, even after removing these low-relief hardbottom sites from the analysis, mean coral cover on Barbuda was only 3.6 ± 0.4%. While past coral cover in Barbuda may not have been high by historical Caribbean standards, where many reefs may have had greater than 50% live coral cover [1,2], we observed areas with abundant standing dead coral skeletons, usually *A*. *palmata* on patch reefs. In these higher relief areas, we are confident that coral cover in Barbuda was greater in the recent past than it is now, because loss of architectural complexity can occur relatively rapidly following coral mortality [58]. However, we have no information about when or why the most recent coral mortality event occurred on Barbuda, nor what the status was in the years prior.

#### Lagoon

We observed 44 different species of fish in Barbuda’s Codrington Lagoon. Because most individuals were juveniles, we report abundance as numerical density rather than in terms of biomass. The most common fishes in the lagoon were yellowfin mojarra (*Gerres cinereus*; 80% of sites; average of 20 ± 6.8 per 100 m^2^), gray snapper (*Lutjanus griseus*; 68% of sites; average of 5.8 ± 1.6 per 100 m^2^) and schoolmaster snapper (*Lutjanus apodus*; 64% of sites; average of 17 ± 4.3 per 100 m^2^; S8 Fig). Schoolmaster were only observed in the channel at the entrance to the lagoon, and gray snapper were most abundant in the channel. In the lagoon, lobsters were most common in the channel, but they were also present in high numbers at one middle lagoon site with a number of ledges and overhangs. Not surprisingly, the vast majority of individual fish and lobsters were juveniles; we observed only one schoolmaster >20 cm (~0.1% of all individuals), and only 19 gray snapper >20 cm (8% of all individuals). We observed no legal lobsters (i.e., >95 mm CL) in the lagoon.

From our observations, the mangrove forests in Codrington Lagoon appeared ecologically intact, with few if any signs of anthropogenic impacts on the mangrove trees themselves. Fast growing fleshy algae, indicators of higher nutrients, were relatively low in abundance in the lagoon, which we interpreted as a positive sign [59]. Mean cover of seagrass was 32 ± 3.8%, and mean cover of seagrass plus calcareous green algae was 45 + 3.6%. Mean cover of macroalgae was 28 ± 3.4% (S9 Fig). In some areas, particularly mangrove areas in the channel, juvenile fish were abundant and juvenile lobsters were common. However, there were some negative indicators in the lagoon. Propeller scars, areas where boat propellers have hit the substrate and removed seagrass, were common in the seagrass beds; these scars can require many years to heal fully, and continued scarring can degrade seagrass beds [60]. From our conversations with locals, *Cassiopeia* jellyfish have dramatically increased in abundance in recent years, but it is unknown what causes these population increases, nor how they may impact local systems.

## Conclusions

Barbuda’s marine ecosystems likely have the potential to support sustainable fisheries over the long term. Based on our conversations with many Barbudans, which were corroborated by our survey data, the abundance of lobsters, conch, fish, and corals are not high compared to other locations in the Caribbean and may be lower than they have been in the past. Fishers report having to go further from port and to deeper waters to make fishing trips worthwhile. Many other Caribbean islands have experienced similar declines but have failed to act, with the result that their fisheries, and in some cases entire ecosystems, have collapsed [1,3,4,6,12,41,45,61].

Currently, several components of Barbuda’s marine ecosystems appear degraded even relative to other locations in the Caribbean, particularly live coral cover [1]. However, recent management actions, such as the implementation of MPAs and a ban on fishing parrotfish, provide reasons to be hopeful. There are still many juvenile lobsters in the reefs and lagoon, and large lobsters—those with the greatest reproductive potential—are still present around the island. Conch are not abundant around Barbuda, but juveniles are still common in some locations. Enforcement of the new MPAs and existing fishery regulations should help increase the reproductive potential around Barbuda, the number and size of fishable adults, and the sustainability of these fisheries. Increasing monitoring of the fisheries and *in situ* populations will provide information to further improve management.

Fish and corals, two of the most commonly measured ecosystem components in the Caribbean, seem to be faring less well than conch and lobster. There are few large predatory or herbivorous fish, both of which are important for local fisheries and may be critical in maintaining ecosystem processes [10,14,21,54]. Coral cover is extremely low in all habitats. However, Barbuda has a relatively small human population and little rainfall and runoff, such that many of the land-based threats impacting other Caribbean islands are likely minimized around Barbuda. This suggests that recent declines in coral cover around the island may be partly the result of indirect effects of fishing—which the governing council of Barbuda may be able to control—or more regional or global factors, such as bleaching and disease, which are beyond local control.

Finally, mangroves and seagrasses, both of which form critical juvenile habitat for many different species, are extensive and healthy in Codrington Lagoon. Juvenile fish and lobsters are locally abundant, especially in the channel. Enforcing the regulations that prohibit destruction of mangroves and seagrasses will help ensure that these habitats remain healthy into the future. Closing Codrington Lagoon to fishing will most likely help increase population sizes of many of these species by allowing juveniles to reach maturity and reproduce before being captured, thereby increasing the size of fishable adult populations.

Following the conclusion of our surveys, the Waitt Institute used these findings in their work with community members and policymakers of Barbuda and in August 2014, the Barbuda Council signed into law comprehensive new ocean management. These new laws included establishing one-third of the coastal waters (and approximately one-third of each habitat type) as protected areas across four marine reserves. Fishing has been temporarily prohibited in Codrington Lagoon, a key nursey habitat, and the Barbuda Council may consider making this protection permanent. Catch of parrotfish was banned island-wide. With enforcement of these new regulations, along with enforcement of existing size restrictions on conch and lobsters, Barbuda’s government met the international target of protecting >30% of territorial waters [62].

Unfortunately, Barbuda took a direct hit from Category 5 Hurricane Irma in September 2017. This hurricane destroyed or damaged the majority of buildings on the island, and displaced all residents. Recovery of infrastructure and the economy will likely take years. The damage to marine ecosystems is unknown, but was also likely substantial. Despite these setbacks, commitments from the Barbuda Council have given the coastal ecosystems of Barbuda a new potential for recovery, resilience, and long-term sustainability.

## Supporting information

S1 Fig Typical reef habitats common around Barbuda. (A, B) Low-relief carbonate flat forereef habitat (C, D) Higher-relief patch reef habitat with standing dead coral.
(TIF)

S2 Fig Abundance of lobsters around Barbuda. (A) sublegal (<95mm carapace length) and (B) legal (>95 mm carapace length) lobsters. Values are the number of lobsters seen at each site.
(TIF)

S3 Fig Abundance of conch around Barbuda. (A) subadult conch (i.e., conch lacking a flared lip) and (B) adult conch (i.e., conch with a flared lip). Values are in numbers per 100 m^2^.
(TIF)

S4 Fig Fish community structure around Barbuda. NMDS plot of density of fish species present at >10% of sites. CONT is continuous reef and ISOL is patch reef. LR, MR, and HR are low (<1.5 m), medium (1.5–3 m), and high (>3 m) relief. Depth categories are shallow (<6 m), mid (6–18 m), and deep (>18 m).
(TIF)

S5 Fig Biomass of key reef fish families around Barbuda. (A) groupers (Serranidae), (B) snappers (Lutjanidae), (C) parrotfish (Labridae: Scarinae), and (D) surgeonfish (Acanthuridae). Values are in g per m^2^.
(TIF)

S6 Fig Pairwise scatterplots of key benthic and fish groups. Values for benthic groups (coral, cca [= crustose coralline algae], turf, and macroalgae) are in percent cover, while values for fish groups (tot_biomass [= total fish biomass] and parrot_biomass [= parrotfish biomass]) are in g per m^2^. Top panels show data and bottom panels show r^2^ and p-values for each pairwise comparison. Note that with 15 comparisons, the significant p-value for α = 0.05 is p = 0.0033.
(TIF)

S7 Fig Live coral cover around Barbuda. Values represent percent benthic cover of live coral.
(TIF)

S8 Fig Abundance of key species in Codrington Lagoon. Shown are the abundance of (A) mojarra, (B) schoolmaster snapper, (c) gray snapper, and (D) lobster at sites surveyed in Codrington Lagoon. Values are number of individuals per 100 m^2^.
(TIF)

S9 Fig Benthic cover in Codrington Lagoon. Frequency distributions of major benthic groups present in Codrington Lagoon.
(TIF)

S1 Table Mean biomass of fish species. Values are mean biomass of fish species from reef fish surveys, in g per m^2^.
(PDF)
