# Supplementary figures and images for: Ecological assessment of the marine ecosystems of Barbuda, West Indies: Using rapid scientific assessment to inform ocean zoning and fisheries management
Source: PLoS One. 2018 Jan 8;13(1):e0189355. doi: 10.1371/journal.pone.0189355 (PMC5757985; doi:10.1371/journal.pone.0189355)

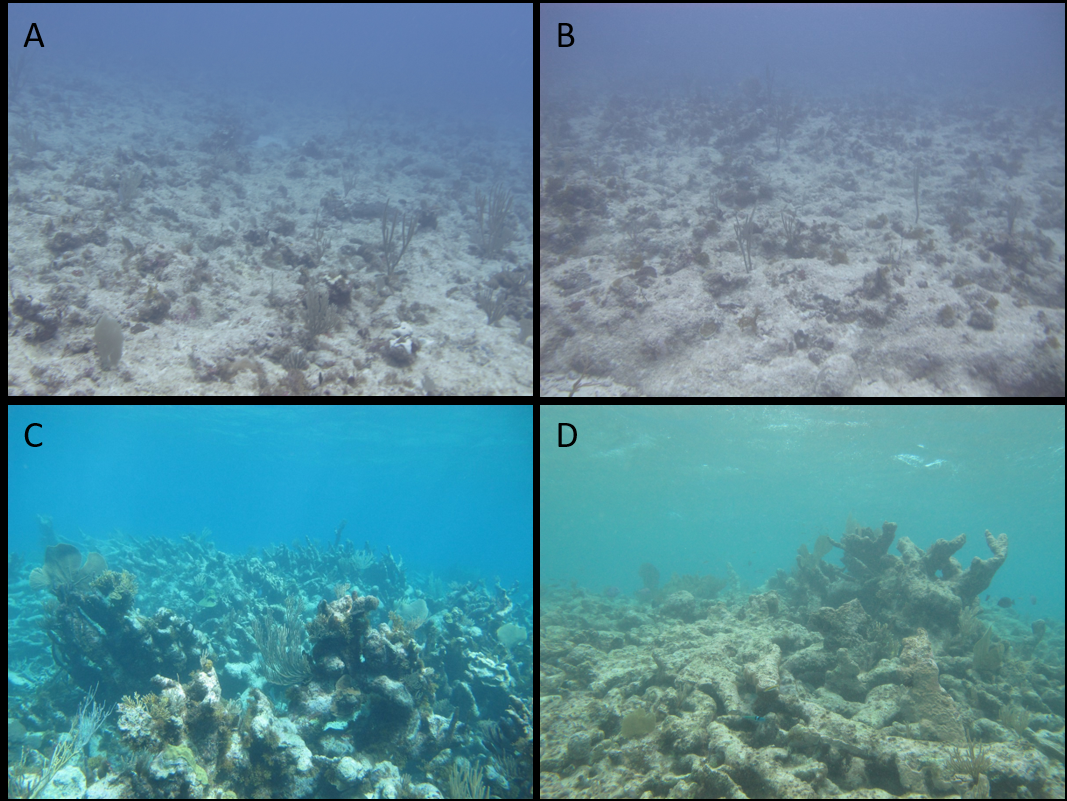

Supplement: S1 Fig — (A, B) Low-relief carbonate flat forereef habitat (C, D) Higher-relief patch reef habitat with standing dead coral. (TIF) [file pone.0189355.s001.tif]

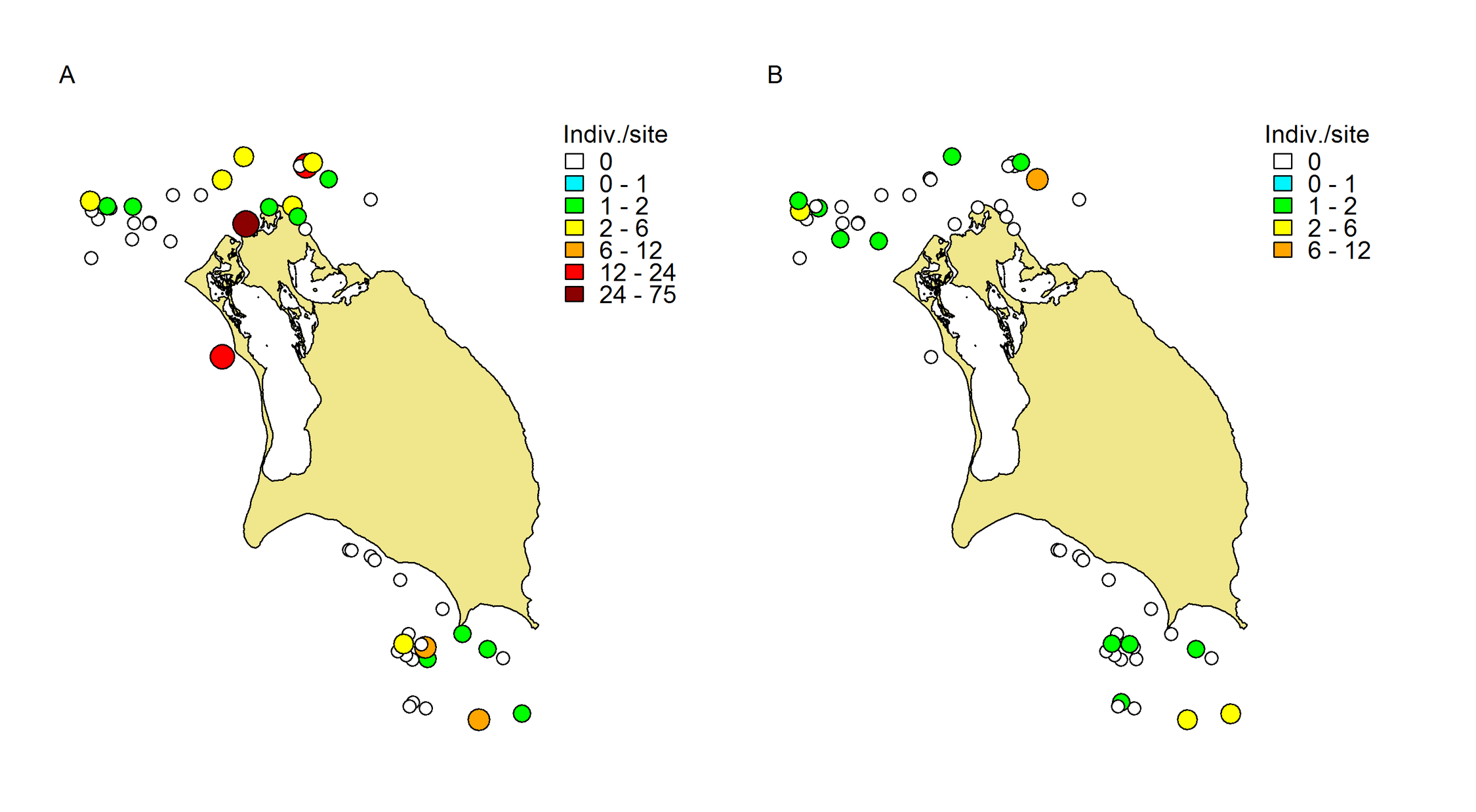

Supplement: S2 Fig — (A) sublegal (<95mm carapace length) and (B) legal (>95 mm carapace length) lobsters. Values are the number of lobsters seen at each site. (TIF) [file pone.0189355.s002.tif]

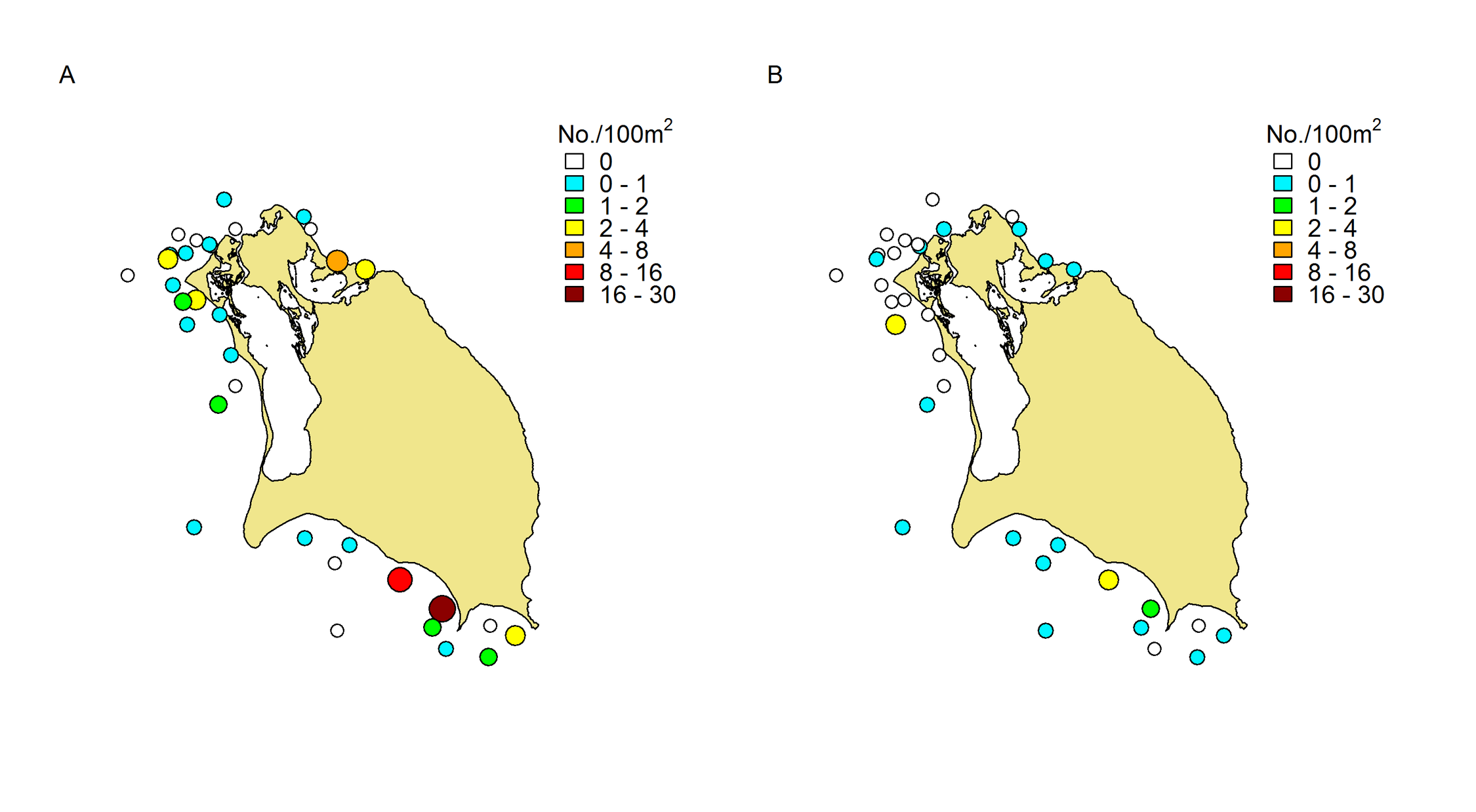

Supplement: S3 Fig — (A) subadult conch (i.e., conch lacking a flared lip) and (B) adult conch (i.e., conch with a flared lip). Values are in numbers per 100 m2. (TIF) [file pone.0189355.s003.tif]

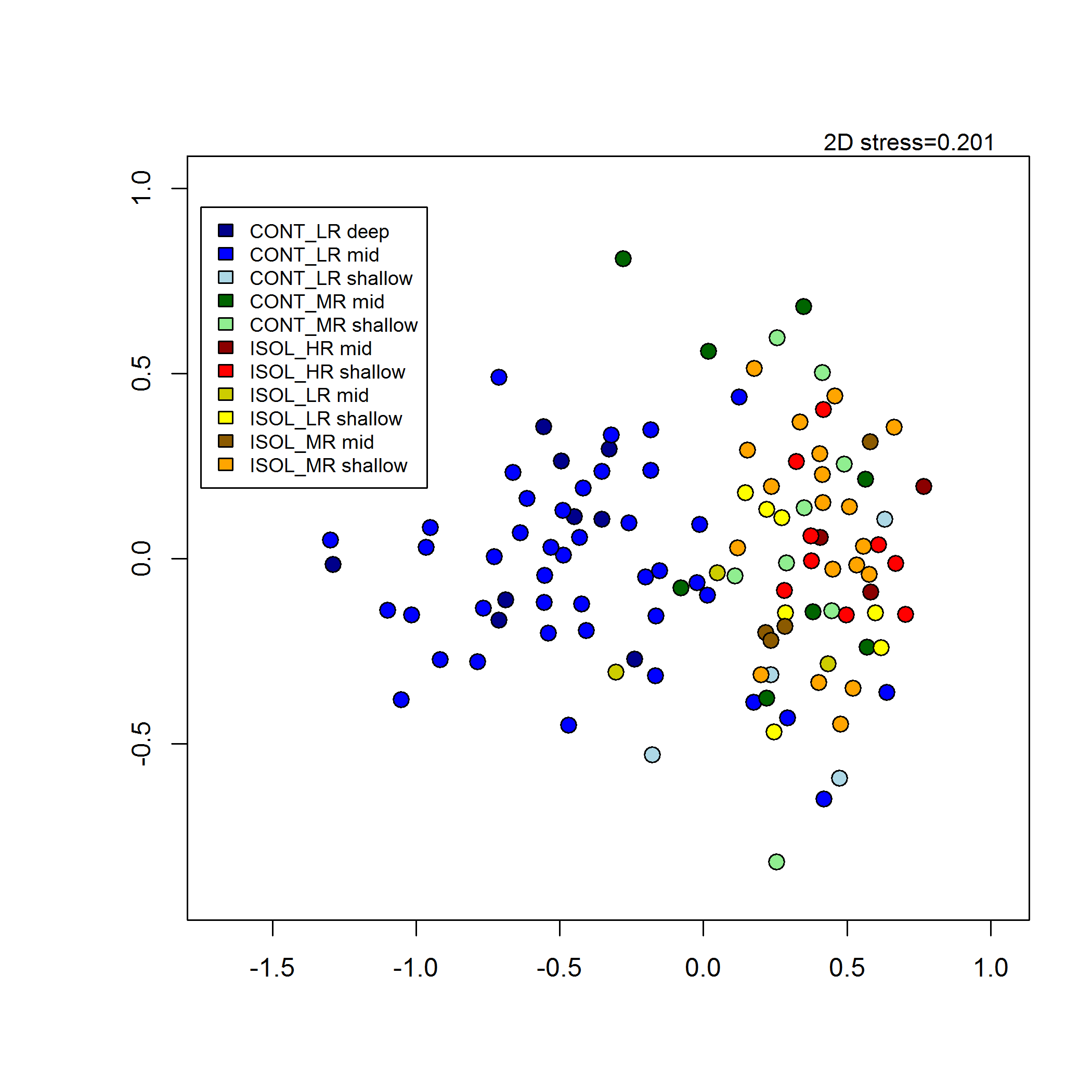

Supplement: S4 Fig — NMDS plot of density of fish species present at >10% of sites. CONT is continuous reef and ISOL is patch reef. LR, MR, and HR are low (<1.5 m), medium (1.5–3 m), and high (>3 m) relief. Depth categories are shallow (<6 m), mid (6–18 m), and deep (>18 m). (TIF) [file pone.0189355.s004.tif]

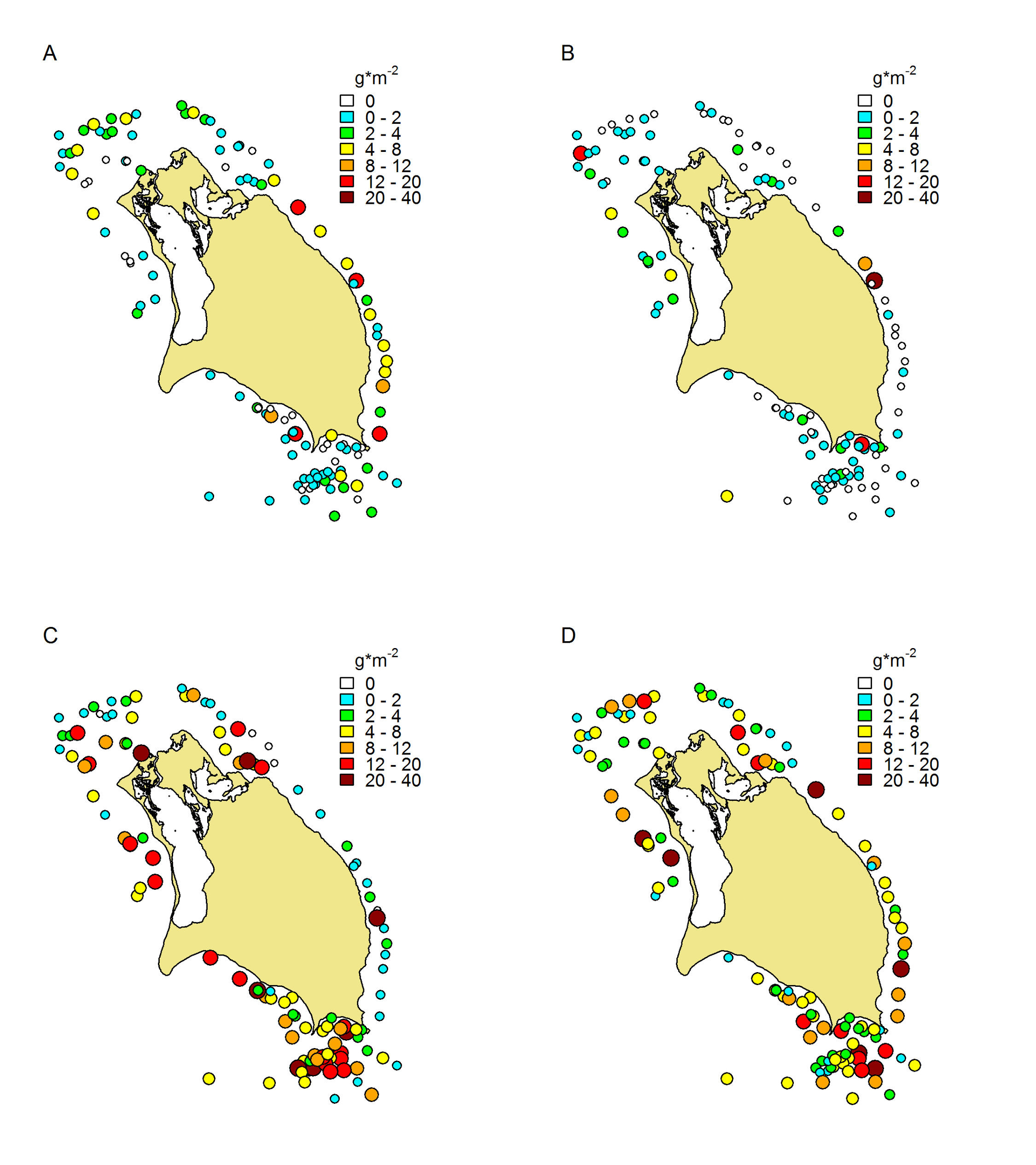

Supplement: S5 Fig — (A) groupers (Serranidae), (B) snappers (Lutjanidae), (C) parrotfish (Labridae: Scarinae), and (D) surgeonfish (Acanthuridae). Values are in g per m2. (TIF) [file pone.0189355.s005.tif]

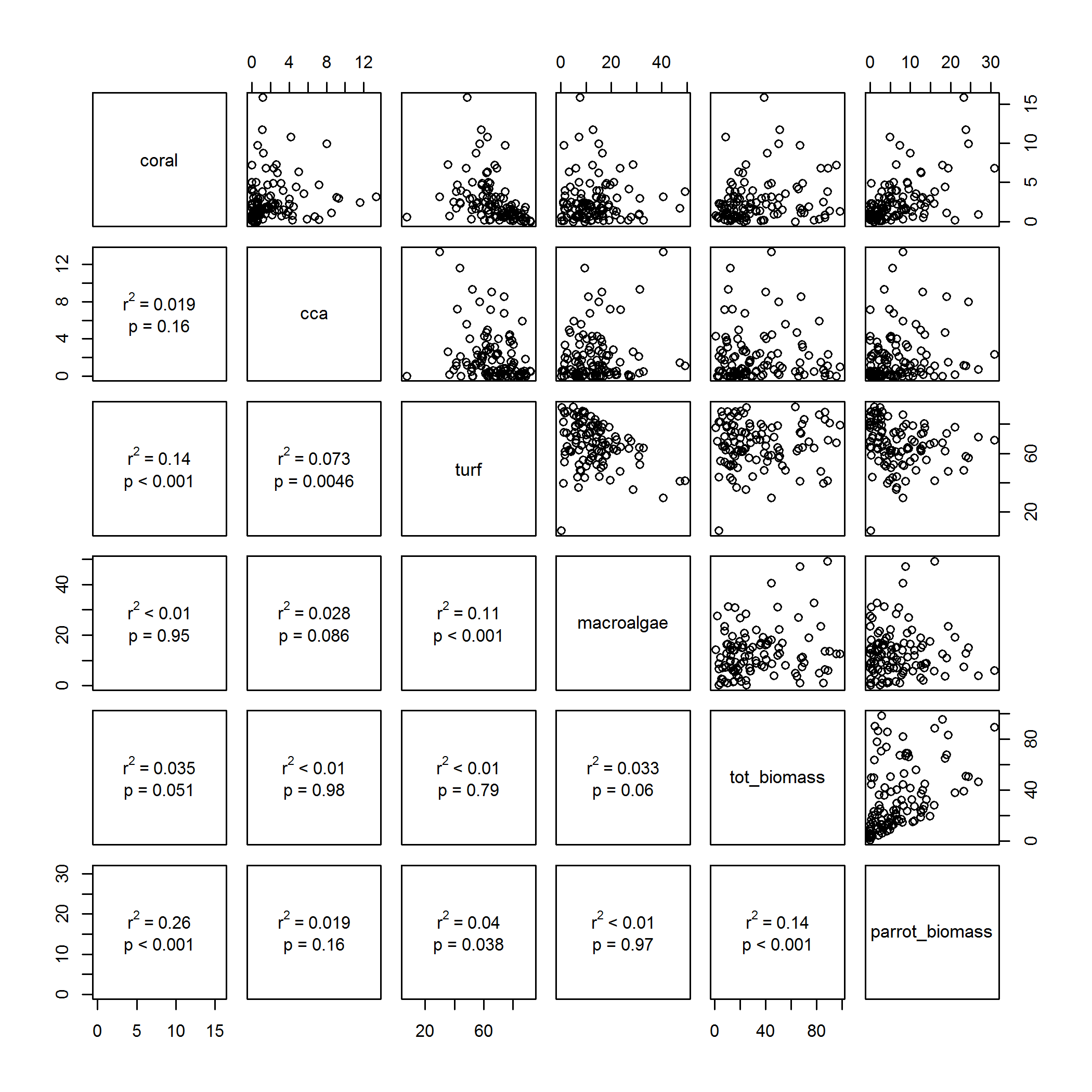

Supplement: S6 Fig — Values for benthic groups (coral, cca [= crustose coralline algae], turf, and macroalgae) are in percent cover, while values for fish groups (tot_biomass [= total fish biomass] and parrot_biomass [= parrotfish biomass]) are in g per m2. Top panels show data and bottom panels show r2 and p-values for each pairwise comparison. Note that with 15 comparisons, the significant p-value for α = 0.05 is p = 0.0033. (TIF) [file pone.0189355.s006.tif]

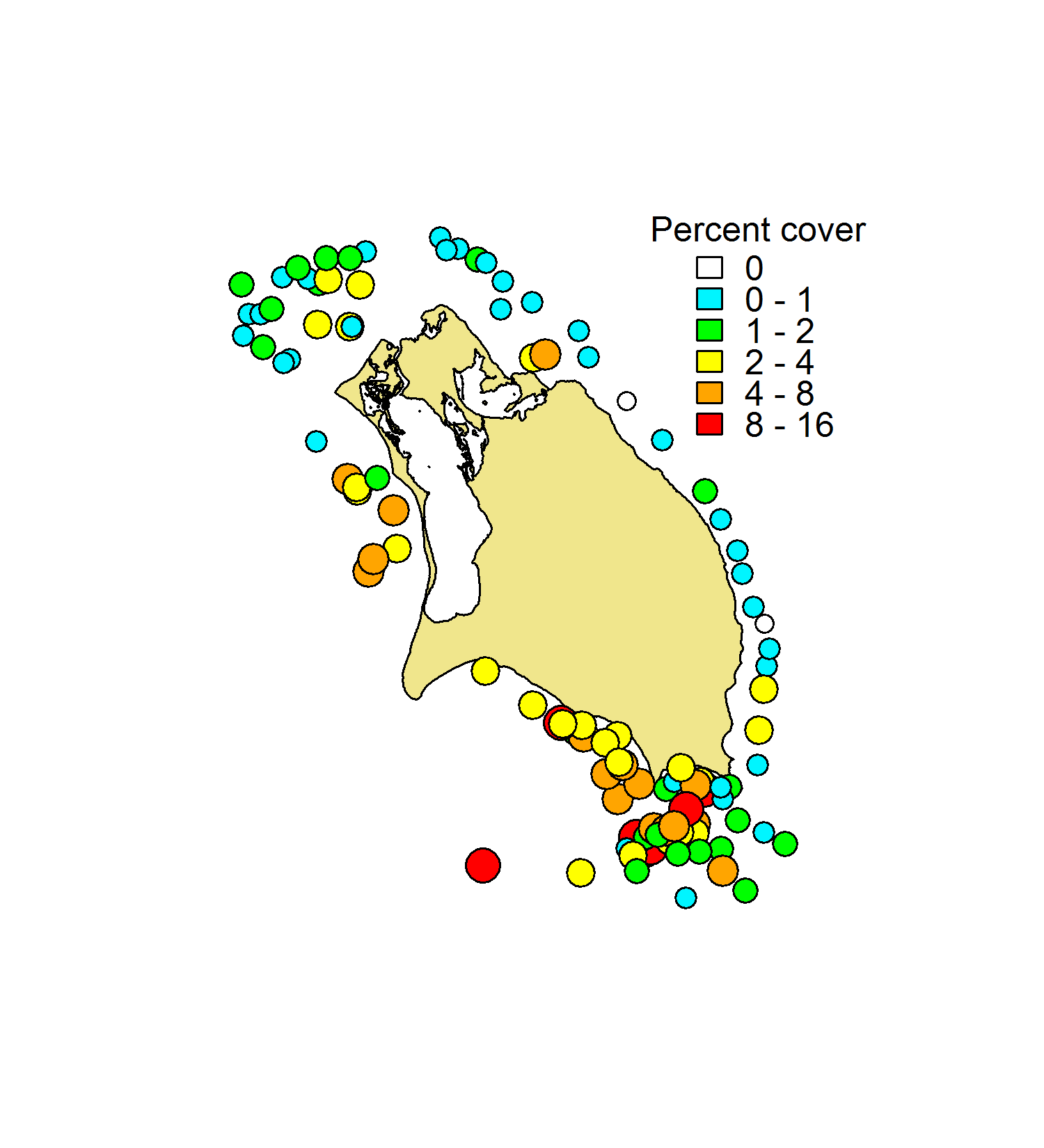

Supplement: S7 Fig — Values represent percent benthic cover of live coral. (TIF) [file pone.0189355.s007.tif]

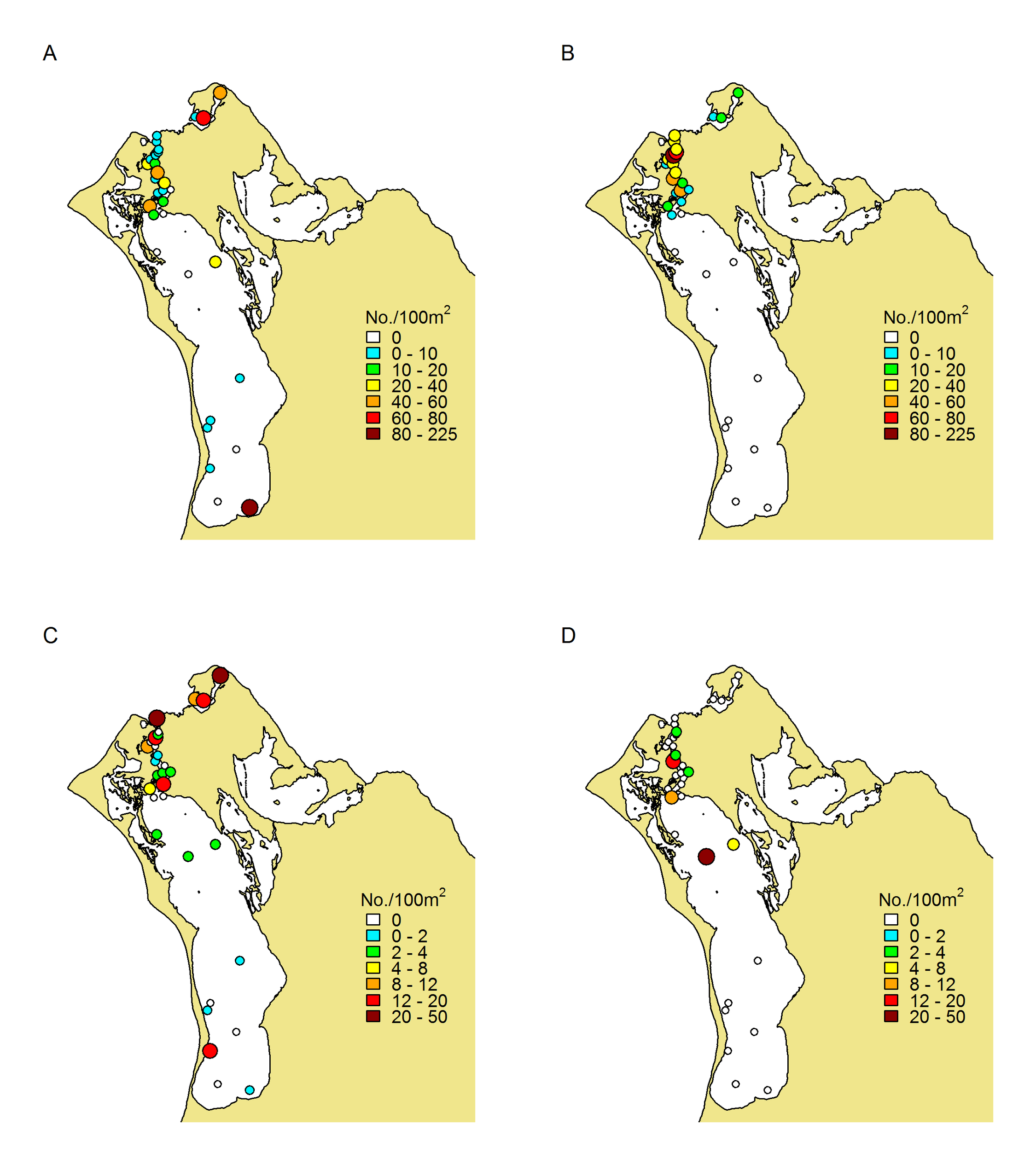

Supplement: S8 Fig — Shown are the abundance of (A) mojarra, (B) schoolmaster snapper, (c) gray snapper, and (D) lobster at sites surveyed in Codrington Lagoon. Values are number of individuals per 100 m2. (TIF) [file pone.0189355.s008.tif]

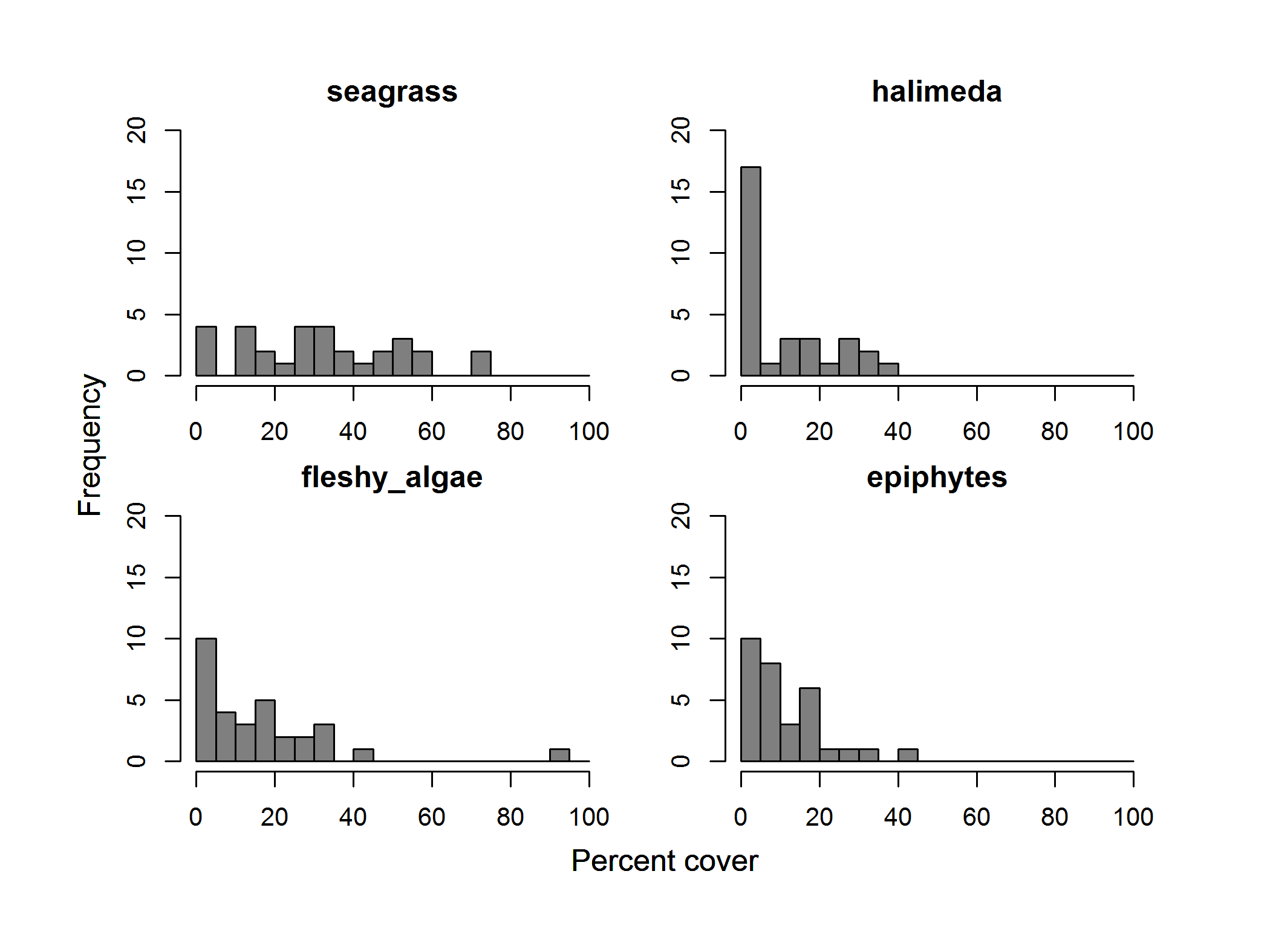

Supplement: S9 Fig — Frequency distributions of major benthic groups present in Codrington Lagoon. (TIF) [file pone.0189355.s009.tif]
