# Supplementary material for: Ecological assessment of the marine ecosystems of Barbuda, West Indies: Using rapid scientific assessment to inform ocean zoning and fisheries management
Source: PLoS One. 2018 Jan 8;13(1):e0189355. doi: 10.1371/journal.pone.0189355 (PMC5757985; doi:10.1371/journal.pone.0189355)
Supplement: S1 Table — Values are mean biomass of fish species from reef fish surveys, in g per m2. (PDF) [file pone.0189355.s010.pdf]

S1 Table.

Species Name	Family	Common Name	Biomass (g*m-2)
Sphyraena barracuda	Sphyraenidae	Great barracuda	2.2343
Melichthys niger	Balistidae	Black durgon	1.3052
Balistes vetula	Balistidae	Queen triggerfish	1.2713
Kyphosus sectatrix	Kyphosidae	Bermuda chub	1.0187
Caranx crysos	Carangidae	Blue runner	0.8410
Aetobatus narinari	Myliobatidae	Spotted eagle ray	0.7528
Decapterus macarellus	Carangidae	Mackerel scad	0.7481
Acanthurus coeruleus	Acanthuridae	Blue tang	0.6125
Holocentrus rufus	Holocentridae	Longspine squirrelfish	0.5770
Caranx ruber	Carangidae	Bar jack	0.5685
Lutjanus analis	Lutjanidae	Mutton snapper	0.5516
Cephalopholis fulva	Serranidae	Coney	0.5131
Sparisoma viride	Scarinae	Stoplight parrotfish	0.4613
Epinephelus guttatus	Serranidae	Red hind	0.4412
Acanthurus bahianus	Acanthuridae	Ocean surgeon	0.4405
Pareques acuminatus	Sciaenidae	High-Hat	0.4301
Acanthurus chirurgus	Acanthuridae	Doctorfish	0.4290
Gerres cinereus	Gerreidae	Yellowfin mojarra	0.4211
Holocentrus adscensionis	Holocentridae	Squirrelfish	0.3199
Epinephelus striatus	Serranidae	Nassau grouper	0.3126
Ocyurus chrysurus	Lutjanidae	Yellowtail snapper	0.2951
Haemulon sciurus	Haemulidae	Bluestriped grunt	0.2937
Sparisoma rubripinne	Scarinae	Yellowtail parrotfish	0.2613
Scarus vetula	Scarinae	Queen parrotfish male	0.2417
Sparisoma aurofrenatum	Scarinae	Redband parrotfish	0.2387
Haemulon flavolineatum	Haemulidae	French grunt	0.2365
Mulloidichthys martinicus	Mullidae	Yellow goatfish	0.2299
Clepticus parrae	Labridae	Creole wrasse	0.2254
Scarus iseri	Scarinae	Striped parrotfish	0.1869
Abudefduf saxatilis	Pomacentridae	Sergeant major	0.1587
Microspathodon chrysurus	Pomacentridae	Yellowtail damselfish	0.1553
Haemulon plumieri	Haemulidae	White grunt	0.1339
Cephalopholis cruentata	Serranidae	Graysby	0.1339
Lutjanus griseus	Lutjanidae	Gray snapper	0.1188
Scarus taeniopterus	Scarinae	Princess parrotfish male	0.1044
Aulostomus maculatus	Aulostomidae	Atlantic trumpetfish	0.0924
Malacanthus plumieri	Malacanthidae	Sand tilefish	0.0910
Lutjanus apodus	Lutjanidae	Schoolmaster	0.0764
Bodianus rufus	Labridae	Spanish hogfish	0.0763
Myripristis jacobus	Holocentridae	Blackbar soldierfish	0.0691

Holacanthus tricolor	Pomacanthidae	Rock beauty	0.0675
Cantherhines macrocerus	Monacanthidae	Whitespotted filefish	0.0481
Stegastes planifrons	Pomacentridae	Threespot damselfish	0.0476
Chaetodon striatus	Chaetodontidae	Banded butterflyfish	0.0465
Scarus guacamaia	Scarinae	Rainbow parrotfish	0.0431
Lactophrys triqueter	Ostraciidae	Smooth trunkfish	0.0393
Pseudupeneus maculatus	Mullidae	Spotted goatfish	0.0379
Pomacanthus arcuatus	Pomacanthidae	Gray angelfish	0.0376
Thalassoma bifasciatum	Labridae	Bluehead wrasse	0.0368
Stegastes leucostictus	Pomacentridae	Beaugregory	0.0328
Caranx bartholomaei	Carangidae	Yellow jack	0.0325
Halichoeres bivittatus	Labridae	Slippery dick	0.0319
Lutjanus synagris	Lutjanidae	Lane snapper	0.0313
Haemulon album	Haemulidae	Margate	0.0312
Diodon hystrix	Diodontidae	Porcupinefish	0.0309
Stegastes adustus	Pomacentridae	Dusky damselfish	0.0273
Chromis cyanea	Pomacentridae	Blue chromis	0.0268
Cantherhines pullus	Monacanthidae	Orangespotted filefish	0.0267
Halichoeres garnoti	Labridae	Yellowhead wrasse	0.0251
Halichoeres maculipinna	Labridae	Clown wrasse	0.0230
Holacanthus ciliaris	Pomacanthidae	Queen angelfish	0.0226
Haemulon carbonarium	Haemulidae	Caesar grunt	0.0220
Sparisoma chrysopterum	Scarinae	Redtail parrotfish male	0.0218
Stegastes partitus	Pomacentridae	Bicolor damselfish	0.0200
Pterois volitans	Scorpaenidae	Red lionfish	0.0197
Bothus lunatus	Bothidae	Peacock flounder	0.0192
Chromis multilineata	Pomacentridae	Brown chromis	0.0185
Heteropriacanthus cruentatus	Priacanthidae	Glasseye snapper	0.0165
Synodus intermedius	Synodontidae	Sand diver	0.0160
Halichoeres radiatus	Labridae	Puddingwife	0.0152
Haemulon aurolineatum	Haemulidae	Tomtate	0.0132
Scomberomorus regalis	Scombridae	Cero	0.0104
Stegastes diencaeus	Pomacentridae	Longfin damselfish	0.0101
Canthigaster rostrata	Tetraodontidae	Sharpnose puffer	0.0087
Haemulon melanurum	Haemulidae	Cottonwick	0.0070
Hypoplectrus puella	Serranidae	Barred hamlet	0.0069
Pomacanthus paru	Pomacanthidae	French angelfish	0.0067
Halichoeres poeyi	Labridae	Blackear wrasse	0.0064
Calamus calamus	Sparidae	Saucereye porgy	0.0060
Scorpaena plumieri	Scorpaenidae	Spotted scorpionfish	0.0047
Lactophrys trigonus	Ostraciidae	Trunkfish	0.0047
Rypticus maculatus	Serranidae	Whitespotted soapfish	0.0046
Halichoeres pictus	Labridae	Rainbow wrasse	0.0046

Halichoeres cyanocephalus	Labridae	Yellowcheek wrasse	0.0034
Hypoplectrus nigricans	Serranidae	Black hamlet	0.0034
Hypoplectrus (species)	Serranidae	Hamlet (species)	0.0030
Triggerfish (species)	Balistidae	Triggerfish (species)	0.0029
Stegastes variabilis	Pomacentridae	Cocoa damselfish	0.0029
Serranus tigrinus	Serranidae	Harlequin bass	0.0028
Chaetodon capistratus	Chaetodontidae	Foureye butterflyfish	0.0027
Sargocentron vexillarium	Holocentridae	Dusky squirrelfish	0.0025
Sparisoma atomarium	Scarinae	Greenblotch parrotfish	0.0019
Neoniphon marianus	Holocentridae	Longjaw squirrelfish	0.0015
Equetus punctatus	Sciaenidae	Spotted drum	0.0014
Hypoplectrus guttavarius	Serranidae	Shy hamlet	0.0013
Sargocentron coruscum	Holocentridae	Reef squirrelfish	0.0012
Lutjanus mahogoni	Lutjanidae	Mahogany snapper	0.0011
Xyrichtys splendens	Labridae	Green razorfish	0.0008
Opistognathus aurifrons	Opistognathidae	Yellowhead jawfish	0.0007
Diodon holocanthus	Diodontidae	Balloonfish	0.0007
Hypoplectrus unicolor	Serranidae	Butter hamlet	0.0005
Sphoeroides spengleri	Tetraodontidae	Bandtail puffer	0.0003
Amblycirrhitus pinos	Cirrhitidae	Redspotted hawkfish	0.0003
Monacanthus tuckeri	Monacanthidae	Slender filefish	0.0003
Serranus tabacarius	Serranidae	Tobaccofish	0.0003
Anchoa lyolepis	Engraulidae	Dusky anchovy	0.0003
Serranus baldwini	Serranidae	Lantern bass	0.0002
Gramma loreto	Grammatidae	Fairy basslet	0.0002
Hypoplectrus (hybrid)	Serranidae	Hybrid hamlet	0.0002
Sparisoma radians	Scarinae	Bucktooth parrotfish	0.0001
